# Eliminating a barrier: Aiming at VISTA, reversing MDSC-mediated T cell suppression in the tumor microenvironment

**DOI:** 10.1016/j.heliyon.2024.e37060

**Published:** 2024-08-30

**Authors:** Yayuan Deng, Mengjia Shi, Lin Yi, Muhammad Naveed Khan, Zhijia Xia, Xiaosong Li

**Affiliations:** a The First College of Clinical Medicine, Chongqing Medical University, Chongqing, China; b Clinical Molecular Medicine Testing Center, The First Affiliated Hospital of Chongqing Medical University, Chongqing, 400016, China; c Department of General, Visceral, and Transplant Surgery, Ludwig-Maximilians-University Munich, Munich, 81377, Germany; d Western(Chongqing) Collaborative Innovation Center for Intelligent Diagnostics and Digital Medicine, Chongqing National Biomedicine Industry Park, No. 28 Gaoxin Avenue, High-tech Zone, Chongqing, 401329, China

**Keywords:** VISTA, MDSC, Tumor microenvironment, ICIs, Immunotherapy resistance

## Abstract

Immune checkpoint inhibitors (ICIs) have revolutionized cancer treatment by producing remarkable clinical outcomes for patients with various cancer types. However, only a subset of patients benefits from immunotherapeutic interventions due to the primary and acquired resistance to ICIs. Myeloid-derived suppressor cells (MDSCs) play a crucial role in creating an immunosuppressive tumor microenvironment (TME) and contribute to resistance to immunotherapy. V-domain Ig suppressor of T cell activation (VISTA), a negative immune checkpoint protein highly expressed on MDSCs, presents a promising target for overcoming resistance to current ICIs. This article provides an overview of the evidence supporting VISTA's role in regulating MDSCs in shaping the TME, thus offering insights into how to overcome immunotherapy resistance.

## 1 Introduction

The advent of immunotherapies has revolutionized the approach to treating cancer by augmenting natural immune responses to tumors [1,2]. Among these therapies, immune-checkpoint blockade is particularly promising due to its remarkable long-term clinical outcomes in advanced-stage cancer patients [3]. Specifically, immune checkpoint inhibitors (ICIs) that target inhibitory immune-checkpoint receptors such as cytotoxic T lymphocyte antigen 4 (CTLA-4), programmed cell death 1 (PD-1), and programmed cell death 1 ligand 1 (PD-L1) have been approved by the Food and Drug Administration (FDA) for treating over nineteen cancer types and two tissue-agnostic conditions [4,5]. However, despite the considerable success of immune checkpoint therapy (ICT), some patients with various tumor types have not benefited from it and may have even experienced life-threatening side effects, known as immune-related adverse events (irAEs), such as cardiac toxicity and acute kidney injury [1,6]. The response to immune checkpoint blockade is influenced by the tumor microenvironment (TME), which often exhibits immune-based resistance [7]. Myeloid-derived suppressor cells (MDSCs) are a critical driver of the immunosuppressive microenvironment in TME and may contribute to primary and acquired resistance to ICIs [8]. MDSCs originate from immature myeloid progenitor cells (IMPs) in chronic pathological conditions like cancer and can suppress the function of T and natural killer cells that fight against tumors [9,10]. Therefore, current research aims to identify new immune checkpoint targets beyond PD-1 and CTLA-4 to overcome resistance [11].

VISTA, also known as PD-1 homolog (PD-1H), differentiation of embryonic stem cells 1 (Dies1), DD1a, Gi24, SISP1, B7-H5, and C10orf54, is an emerging immune checkpoint protein that promotes cancer progression by suppressing immune responses, thereby making VISTA a promising target for cancer immunotherapy [12,13]. Previous studies suggest that VISTA shares homology with members of the B7 family but displays unique biological properties. VISTA regulates both innate and adaptive immune responses and can serve as both a ligand and a receptor in intercellular communication, although its function remains to be fully elucidated [12,14]. VISTA is highly expressed in tumoral cells, lymphoid cells, and hematopoietic cells. VISTA overexpression has been observed in several tumor types, including small intestine cancer, stomach cancer, pancreatic cancer, sarcoma, and neuroendocrine tumors, all of which are more than the average proportion across all cancers [15]. Unlike other negative checkpoint regulators that are expressed on activated T cells, VISTA is expressed on naive T cells and helps maintain their quiescence and peripheral tolerance under steady-state conditions [16]. Notably, myeloid cells exhibit the highest levels of VISTA expression [17]. In fact, VISTA expression is tenfold higher in MDSCs in the TME compared to peripheral lymph nodes [18]. Based on this, several studies have suggested that VISTA may play a crucial role in regulating the MDSC population. However, few reviews have focused on this topic. To address this gap, this review synthesizes relevant studies exploring the relationship between VISTA and MDSCs and examines the potential of VISTA in modulating MDSCs.

## 2 The relationship between VISTA and MDSC

### 2.1 VISTA highly expressing on MDSC

Notably, the myeloid lineage exhibits the greatest levels of VISTA expression, which is primarily expressed within the hematopoietic compartment. The greatest amounts of VISTA protein expression have been observed in MDSCs [14,19]. In the peripheral blood of AML patients, Wang L et al. found that VISTA is strongly expressed on MDSCs [20]. Hypoxia and DNA methylation are two mechanisms that boost VISTA expression on MDSC. Low oxygen levels, or hypoxia, are a characteristic of solid tumors [21] and they cause the increase of VISTA on MDSCs in the TME [22]. The inadequate oxygenation in TME results in a hypoxic state that promotes tumor angiogenesis, tumor development, and spread [23]. This hypoxic state is brought on by the fast growth of tumor cells and the twisted blood vessels. One way that hypoxia invades the TME is by increasing the activity of CXC family members and their corresponding CXC receptors (CXC-R) [24]. On the other hand, in the presence of hypoxia, HIF-1 attaches to conserved hypoxia-responsive regions in the VISTA promoter, upregulating VISTA on MDSCs in the TME [22,25]. The primary hypoxic regulator is HIF-1. Prolyl hydroxylases (PHDs) cannot hydroxylate two proline residues (Proline-402 and Proline-564) in the HIF-1 component in low oxygen environments, and as a result, von Hippel-Lindau protein (pVHL) cannot ubiquitinate its target, stabilizing HIF-1 [25]. Since the downstream gene expression switches cellular metabolism from oxidative phosphorylation to glycolysis, stabilizing (HIF-1) is essential for the adaptive response to hypoxia-induced oxidative stress [26]. Deng et al. discovered using flow analysis that hypoxic (pimo+) CD11b^+^ myeloid cells expressed significantly more VISTA than non-hypoxic cells, and that VISTA was expressed at greater levels on CD11b^high^Gr1^+^ MDSCs (pimo-). Additionally, under normoxic circumstances, CD11b^+^myeloid cells still expressed VISTA mRNA and protein in particular HIF-1 knockdown hPBMCs. Hypoxic antibody targeting or VISTA genetic deletion alleviated MDSC-mediated T-cell repression [22].

DNA methylation is a mediator of VISTA transcriptional control in MDSCs [27]. Through DNA methylation, histone post-translational alterations, and non-coding microRNAs (miRNAs), epigenetics alters phenotype that is not based on DNA structure [28,29]. The role of MDSC is altered epigenetically, which changes its features and reframes the microenvironment to inhibit tumor development and metastasis [30]. DNA methyltransferases (DNMTs) catalyze DNA methylation, and the two DNMT families, DNMT3 and DNMT1, are responsible for methylation establishment and maintenance, respectively [31]. The ten-eleven translocation (TET) enzymes, which function as 5-methylcytosine oxidases, can reverse locus-specific methylation [32]. In CD33^+^HLA-DR^-^ myeloid cells, Saleh et al. discovered that DNMT3a is downregulated while TET enzymes are upregulated. This finding raises the possibility that TET-mediated active demethylation is what is responsible for the increase of genes in CD33^+^HLA-DR^-^MDSCs cells. Additionally, it has been discovered that VISTA mRNA expression levels are similarly upregulated in both CD33^+^HLA-DR^-^ and CD33^+^HLA-DR^+^ MDSC subpopulations, whereas PD-L1, MMP9, and TIM-3 mRNA expression levels are significantly upregulated in the former compared to the latter, which is linked to CpG promoter unmethylation [27].

### 2.2 The structure of VISTA

The Vsir gene in rodents encodes the protein VISTA, which is highly conserved between species [33]. The mouse VISTA gene, which has 930 base pairs and can be transcribed into a type I transmembrane protein with a length of 309 amino acids (aa), is found on the forward strand of chromosome 10 (location 10qB4) [34]. There is 76 % similarity between a single extracellular Ig-V domain with 136 amino acids and a 23 amino acid stalk section and the human protein. Human VISTA and the transmembrane region, which has 21 amino acids and a cytoplasmic tail with 97 amino acids, are 90.6 % identical [34]. The human VISTA gene, like its mouse counterpart, produces a type I transmembrane protein. Human VISTA protein contains a 130-aa extracellular IgV domain, a 33-aa stalk region, a 32-aa signal peptide, a 20-aa transmembrane domain, and a lengthy 96-aa cytoplasmic tail [35]. The H-, A-, G-, F-, C-, and C′-strands make up the front face of the overall framework of VISTA, while the A′-, B-, E−, D-, and C″-strands make up the rear face. Three disulfide links that include each of the six cysteine residues have been established in its ECD according to the VISTA sequence [36]. Although the conserved cytoplasmic tail of VISTA lacks the traditional ITIM/ITAM motif, it has been discovered to contain possible protein kinase C binding sites and a proline-rich motif that may serve as a substrate for interaction [19].

The B7 family member that is most closely related to PD-L1 in terms of development is VISTA [37]. Numerous obvious distinctive characteristics of the VISTA amino acid sequence set it apart from other CD28/B7 family members, according to bioinformatic study [38]. The addition of an extra "H" -strand gives VISTA a lengthier IgV-Like do-main in the length of 137 amino acids compared to other V-set Ig domains, which limits VISTA's orientation on the cell surface [38]. The presumed B and F segments of the VISTA IgV domain are connected by a canonical disulfide link [19]. Additionally, it stands out from other members of the family by having about 20 continuous amino acids in the C-C′ region, 30 intramolecular hydrogen bonding contacts, and a disulfide bond (C51/C113). In contrast, the loop that links the two beta strands in PD-L1 and other B7 family proteins is a much more compact four residues. Additionally, the expanded C-C′ loop's protrusion from the beta-sandwich core is probably stabilized by two non-canonical and conserved disulfide bonds, or the distinctive structures of the VISTA, which link residues C12/C146 and C51/C113, respectively. Additionally, the histidine concentration of VISTA is significantly higher, especially in the FG loop region [36,38]. Its extracellular domain (ECD) contains 8.6 % histidine; in comparison, all type 1 transmembrane ECDs have a mean histidine concentration of only 2.4 % [38]. These distinct histidine groups with open surfaces may be very important for T-cell inhibition [38].

In summary, VISTA exhibits a relationship with other members of the B7 family but also has noticeable differences.

### 2.3 Binding partners for VISTA

**Fig. 1:**
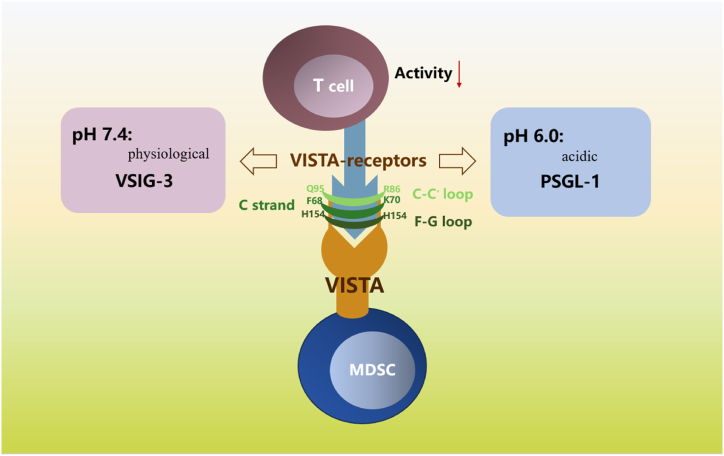
Main biding partners of the VISTA and the involved structures. VISTA interacts with VSIG3 at physiological pH, but acidic (pH∼6.0) tumor microenvironment (TME) facilitates VISTA-expressing cells binding to PSGL-1 on T cell. Both interactions curtail T cell activities. Both associations reduce the activity of T cells [46]. Both engage with VISTA in the F68 and K70 of the C strand, the R86 and Q95 of the C-C′ loop, and the F-G loop (H154) [47].

VISTA serves dual roles as both a protein and a receptor [115]. According to studies, VISTA functions as an agonist for both the T cells' VSIG-3 receptor and the PSGL-1 receptor on T cells (Fig. 1). Other possible ligands for VISTA include VSIG8, NSC622608, and Galectin 9 [[39], [40], [41], [42], [43]].

VSIG3, also referred to as IGSF11, BT-IgSF, BTIGSF, CT119, and CXADRL1, is a 46 kDa protein with 431 amino acids that is a member of the immunoglobulin group [44]. In gastrointestinal malignancies, such as colorectal cancer, hepatocellular carcinoma, and gastric cancer, VSIG3 is heavily expressed. Through its association with VISTA, VSIG3 regulates immune cell activity and influences the anti-tumor immune response.

PSGL-1, a 120 kD transmembrane protein that is predominantly produced as a homodimer on lymphoid and myeloid cells, has long been researched as an adhesion molecule involved in immune cell trafficking and is acknowledged as a regulator of many aspects of immune reactions by myeloid cells [45]. It is conceivable that the PSGL-1/VISTA pathway may play a significant role in preventing T-cell activation in acidic circumstances.

Potentially pH-selective is VISTA [39]. At physiological pH, VISTA engages with VSIG3, but an acidic (pH 6.0) TME makes it easier for VISTA-expressing cells to attach to PSGL-1 on T cells. Both associations reduce the activity of T cells [39,40]. The pH of the tumor microenvironment can therefore likely be targeted in order to decrease the immunoinhibitory activity of the VISTA. In the research by Mehta et al. the binding preferences of VISTA for PSGL-1 and VSIG3 were contrasted. The putative binding affinity for PSGL-1 was 4 nM at pH 6.0, which is frequently found in the TME, while the affinity for VSIG3 was 80 nM. In contrast, VSIG3's binding propensity to 20 nM increased four-fold at pH 7.4 while no evident binding to PSGL-1 was found [46]. Both engage with VISTA in the F68 and K70 of the C strand, the R86 and Q95 of the C-C′ loop, and the F-G loop (H154). Likewise, the nonprotruding F-G loop's three histidine residues, H153, H154, and H155, are crucial for PSGL-1 attachment. The inhibiting function of VISTA in antigen-specific T-cell tests was also decreased by substituting alanines for the triple-histidine cluster [47]. Additionally, under acidic circumstances, the amino group on the extracellular domain of histidine-rich VISTA is protonated, enabling contact with negatively charged PSGL-1. Human CD4^+^ T cells cocultured with VISTA-expressing cells in vitro produce more IFN-γ, have NF-B phosphorylated, and are more likely to proliferate due to an acidic pH-selective VISTA mAb that inhibits the PSGL-1/VISTA interaction [39]. The research by Johnson et al. demonstrates how environment affects lymphocyte-myeloid communication. Lymphocytes and myeloid cells can be harmed by the acidic and anaerobic conditions present in solid tumors [48].

### 2.4 VISTA modulates MDSC-mediated immunosuppression

Cancer cell interactions with their surroundings are crucial for the survival and development of tumors. The extracellular matrix (ECM) and soluble components, such as immune cells, are included in the TME [49].

**Fig. 2:**
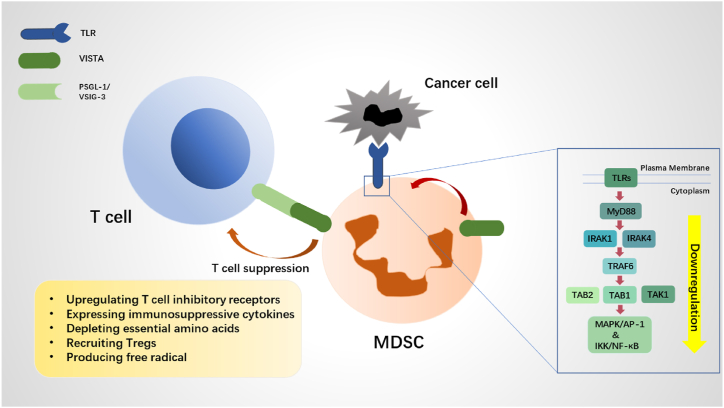
MDSCs create immunosuppressive TME and the TLR/MyD88 signaling on MDSC is downregulated by VISTA. MDSC primarily inhibits T cells by 1) increasing the expression of T cell immunoinhibitory receptors, 2) secreting immunosuppressive cytokines, 3) depriving T cells of critical amino acids for activity and proliferation, 4) gathering Tregs, and 5) enhancing the generation of free radical. The TLR/MyD88-mediated communication is downregulated by VISTA. After TLRs are activated, the MyD88/IRAK1/4 complex recruits and triggers TRAF6, which in turn stimulates the MAPK/AP-1, IKK/NF-B, and MAPK pathways.

Within the TME, myeloid-derived suppressor cells (MDSCs) contribute to an immunosuppressive environment that aids tumor evasion, which is by increasing the expression of T cell immunoinhibitory receptors, secreting immunosuppressive cytokines like IL-10 and TGF-β, depleting essential amino acids for T-cell activity and proliferation, facilitating the development of tumor-specific Tregs, and encouraging the production of free radical like ROS (Fig. 2) [50]. Notably, the elevated expression of VISTA on MDSCs suggests that VISTA may directly control MDSCs' ability to inhibit T cells in hosts with tumors [51].

**Fig. 3:**
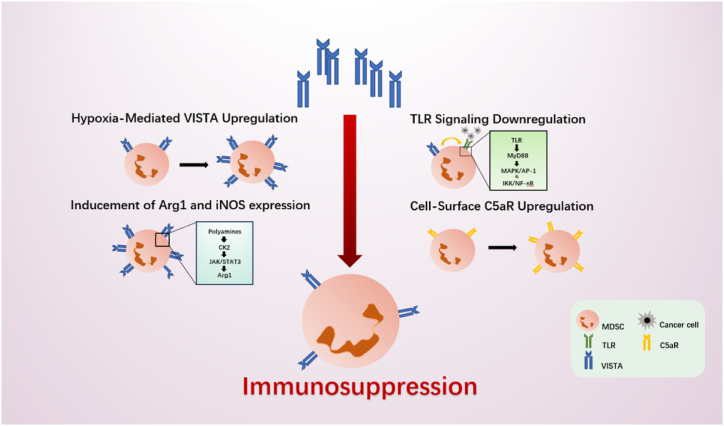
VISTA regulates MDSC-mediated inhibition through various mechanisms. By increasing the expression of VISTA on MDSC, hypoxia improves the delivery of co-inhibitory signals to T cells. VISTA inhibits TLR-mediated communication and the development of the MDSC C5a receptor on cell surfaces. The MDSC-mediated suppression enhancement through ARG1 also involves VISTA.

Hypoxia enhances the inhibitory function of MDSCs in addition to upregulating VISTA on MDSCs in the TME (Fig. 3) [22]. First, since VISTA acts as a negative checkpoint regulator (NCR) by providing co-inhibitory signals upon counter-receptor engagement to reduce T cell response, its upregulation can significantly increase MDSCs' capacity for immunosuppression [52]. Second, hypoxia is one of the processes that helps to create an acidic pH environment, along with oncogene activity, the Warburg effect, and the interaction between VISTA and its ligands or receptors that is pH-selective [47]. HIF-driven glycolytic enzymes like lactate dehydrogenase A (LDHA), glucokinase (GCK), pyruvate kinase M2 (PKM2), and phosphofructokinase 1 (PFK1) were discovered to have dramatically elevated levels in conjunction with the upregulated HIF-1 expression under hypoxic circumstances [53]. A substantial quantity of lactate is produced during glycolysis, which is aided by hypoxia, aggravating the already acidic (pH 6.5–6.7) milieu [23]. The extracellular pH is significantly lowered as a result of the metabolism of the growing tumor cell, which also generates a large number of acidic metabolites and reduces their clearance [54]. Otto Warburg was the first to show that, even under aerobic circumstances, proliferating cancer cells increased their glucose intake and generated lactate. In cancer cells, the Warburg effect lowers the amount of ATP and CO_2_ produced by the mitochondria, which encourages the development of an alkaline internal pH (pHi) and an acidic external pH [55]. TME immunosuppressive niches and probable VISTA activity areas are caused by an acidic pH [47]. At an acidic pH, but not a normal pH, VISTA expressed on cancer cells, MDSCs, or tumor-associated macrophages (TAMs) can attach to PSGL-1 on T cells [47]. In acidic circumstances, VISTA engages with PSGL-1 and sends a signal into the T cell that inhibits immune function [47].

Inducing a more immunosuppressive phenotype in rodents through VISTA signaling on myeloid cells can prevent the myeloid differentiation primary response to Myeloid differentiation factor 88 (MyD88)-dependent Toll-like receptor (TLR) signaling and cytokine secretion (Fig. 3) [47]. TLRs are pattern recognition receptors (PRRs) that detect various molecular sequences linked with pathogens (PAMPs). Following the recognition of PAMPs, PRRs initiate intracellular signaling cascades that result in the increase of co-stimulatory molecules as well as the induction of inflammatory cytokines and chemokines [56]. TLRs thus play a crucial part in controlling the immunological and inflammatory responses of the organism. MyD88-dependent pathway (TLR2/4/5/7/8/9) [57]. In the MyD88-dependent pathway, TLRs associate with TIR domains of MyD88 and MyD88 adaptor-like (Mal), a bridging adaptor between MyD88 and TLRs, recruiting serine/threonine kinases IRAKs (interleukin-1 receptor-associated kinases) to stimulate the TRAF6/IKK complex and MAPKK, which result in activation of MAP kinases, NF-κB and AP-1 [58]. The interleukin-1 receptor-associated kinases (IRAKs) are recruited by TLRs in the MyD88-dependent pathway, where they associate with MyD88's TIR domains and MyD88 adaptor-like (Mal), a bridging adaptor between MyD88 and TLRs. This stimulation of the TRAF6/IKK complex and MAPKK leads to the activation of MAP kinases, NF-B, and AP-1. A classic example of a pro-inflammatory signaling system is the nuclear factor NF-B pathway [59]. Tumor necrosis factor-alpha (TNF-α), interleukin-6 (IL-6), IL-12, IL-1, and adhesion molecules are just a few examples of the cytokines and chemokines that are activated by NF-B after it separates from IB and moves to the nucleus [58]. By encouraging TRAF6 protein breakdown and limiting the stimulation of the MAPKs/AP-1 and IKK/NF-B signaling pathways, VISTA inhibits TLR-mediated signaling (Fig. 2) [13]. Absence of VISTA increases TLR-mediated generation of reactive cytokines. The expression of numerous cytokines and chemokines, such as IL12, IL6, GM-CSF, IFN-, MIP-1, and MIP-1, is substantially enhanced when Vsir/macrophages are stimulated ex vivo with TLR agonists CpG (TLR9) and R848 (TLR7) [51]. Except for PMN-MDSCs, VISTA inhibition increased the secretion of immune-stimulatory cytokines like IL12p40 in M-MDSCs [51]. In reaction to anti-CD3 stimulation, the addition of HMBD-002, an anti-VISTA antibody that blocks the C-C′ loop region of VISTA, effectively reversed MDSC-mediated T cell suppression, as shown by the increased levels of IFN-γ [60]. In low pH circumstances that simulate the TME, the highly selective anti-VISTA antibody PMC-309 binds to MDSC cells' VISTA and boosts the release of IFN-γ, TNF-α, and IL-2 in Mixed Lymphocyte Reaction (MLR) conditions [61]. Similar functions are performed in reversing MDSC immunosuppression by KVA-12.1, a new totally human anti-VISTA antibody [62]. Major immune response regulator IFN-γ, opposes the production of suppressive cytokines (TGF-β, IL-10) and encourages the development of IL-12. Additionally, macrophages' tumorigenic M2-differentiation is suppressed by IFN-γ [63]. Additionally, TLR4, MD-2, and MyD88 were required for the S100A8 (2–89) peptide to induce cell motility. After five days of therapy with Eritoran, a TLR4/MD-2 complex blocker, the recruitment of tumor-infiltrating myeloid suppressor cells was decreased [64]. When compared to MyD88^+/+^ TRAMPTg ^±^ mice, the prostates of MyD88^−/−^ TRAMPTg ^±^ mice had more CD11b^+^ Gr1^+^ myeloid-derived suppressor cells (MDSCs), according to an analysis of invasive immune groups [65]. The MyD88-NF-B signaling pathway's effect on MDSCs, however, is complicated. Prostaglandin E2 from tumors (PGE2) causes nuclear p50 NF-B buildup in M-MDSCs, shifting their response to IFN toward NO-mediated immunosuppression and lowering TNF production [66]. By triggering the JNK-AP1 and Jak2-IRF1 signaling pathways, IFN + LPS significantly increased the production of the iNOS protein [67]. The simultaneous stimulation of ARG and NOS in myeloid cells harboring the tumor can produce strong inhibiting signals that ultimately cause anti-gen-specific T lymphocytes to undergo apoptosis [68]. According to Hu et alstudy, .'s the formation of MDSC was inhibited by NF-B inhibitors, and the growth of MDSC was dependent on the MyD88-NF-B pathway [69]. According to Zhang et al. MDSCs migrate to the tumor location via the CXCL2/MIF-CXCR2 signaling pathway, which in turn triggers the KF-b and MyD88-dependent MAPK pathways in MDSCs. Following MyD88 reduction, CXCR2 expression and p65, ERK, and p38 activation were reduced [70].

Once compared to cells from WT mice, VISTA-KO mice's peripheral blood and bone marrow both contained M-MDSCs and PMN-MDSCs, which reliably displayed lower surface expression of the C5a receptor [71]. The complement system's C5a protein is one of over 30 others [72]. Complement can be activated by DAMPs in the setting of cancer treatment and function as an effector of antibody-mediated tumor cytotoxic reactions. Complement activation, however, also has the potential to change the TME towards a tumor-supportive environment depending on a number of variables, including the tumor's cellular origin, its innate ability to produce autologous complement proteins, the makeup of the tumor microenvironment, and the degree of complement activation [73]. By affecting vascularization, the buildup of aberrant immune cells, and the extracellular matrix proteins, complement activity may have an effect on the development of the pre-metastatic niche [74]. By luring PMN-MDSCs to the main tumor location via its standard receptor C5aR1, C5a inhibits the antitumor CD8^+^ T cell-mediated response [75]. In the PMN-MDSC subgroup, C5a caused an amoeboid-like migration marked by a decrease in the quantity and size of focal adhesions, a reconfiguration of the cytoskeleton, an upregulation of CD11b, and a drop in extracellular 1 and 3 interferons through clathrin-coated endocytosis [74]. In the PMN-MDSC subgroup, C5a caused an amoeboid-like migration marked by a decrease in the quantity and size of focal adhesions, a reconfiguration of the cytoskeleton, an upregulation of CD11b, and a drop in extracellular 1 and 3 interferons through clathrin-coated endocytosis [76]. When C5a is present, M-MDSCs produce more ROS, which cause oxidative stress, which inhibits T cell translation, prevents T cells from adhering to modified MHC, and causes peripheral CD8^+^ T cells to become antigen-specifically tolerant [77]. In conclusion, VISTA may influence MDSC inhibition via C5a.

VISTA enhances M-MDSCs ability to suppress T cell expansion by promoting STAT3 activation, which may induce ARG1, a key enzyme supporting polyamine biosynthesis, under normoxia and certain hypoxia conditions (Fig. 3) [78]. In order to deplete L-arginine required for T cell proliferation, MDSCs exhibit large amounts of ARG1 and iNOS. The two enzymes deplete L-arginine by converting it into L-ornithine, urea, and NO while catabolizing it as a substrate [79]. By inhibiting cyclin D3 and cyclin-dependent kinase 4 (CDK4), L-arginine deprivation stops the T cell cycle in the G0-G1phase [80] and lowers the transcript of the T cell antigen receptor zeta chain (CD3zeta) [81]. In the meantime, NO suppresses T cell function through a variety of mechanisms, including attenuating MHC class II molecule induction, reversibly blocking IL-2 receptor signaling, inhibiting JAK3 and STAT5 function in T cells, mediating T cell DNA damage, and encouraging the differentiation of highly suppressive Treg cells through a cGMP-dependent mechanism [[82], [83], [84], [85], [86]]. It is shown that overexpression of STAT3 mutants (STAT3^Y705E^ and STAT3^S727D^) upregulated AGR1 expression in Vsir^−/−^ MDSCs. Zhang et al. observed that phosphorylation of STAT3^S727^ mediated by ERK1/2 activation is inhibited VISTA deletion. In addition, to sustain their own biosynthesis, polyamines promoted CK2 activity, STAT3 activation, and ARG1 expression in a positive feedback loop. In both WT and Vsir^−/−^ MDSCs, ARG1 expression was diminished by STAT3 inhibitor (Stattic) treatment and rescued by putrescine supplementation. Besides, VISTA established a transcriptomic program that augments cellular metabolism, proliferation, and immunosuppressive function of tumor-associated MDSCs. Gene set enrichment analysis (GSEA) revealed that arginine metabolism, glycolysis, lipid metabolism, serine synthesis and mitochondrial respiration are downregulated in Vsir^−/−^ MDSCs, while antigen presentation (e.g., MHCII, Ciita, CD74, etc.) and inflammatory signaling (e.g., Ifi202b, Grap, the C1q family, the Fc receptor family, Mmp-family genes, etc.) genes are upregulated. The transcriptomic changes in Vsir^−/−^ MDSCs may be the result of not only STAT3-dependent activities but also polyamines that may directly interact with DNA, transcription factors, and chromatin-remodeling proteins [78].

### 2.5 Anti-VISTA contributes to reducing MDSC in TME

Extensive research has validated that VISTA blockade contributes to reducing MDSCs (see Table 1) [22,51,60,87,88].

**Table 1:** Targeting VISTA contributes to reducing MDSCs.

Treatment	Drug name	Tumor model	Predominant MDSC population	MDSC reduction	Publication year	Ref
VISTA monoclonal antibody (mab)	13F3	Murine bladder tumor model MB49	PMN-MDSCs (Cd11b ^+^ Gr1^hi^Ly6G ^+^ Ly6C^−int^)	No obvious reduction	2014	[87]
	13F3	Murine melanoma model B16OVA	M-MDSCs (Cd11b ^+^ Gr1^int^Ly6G^−^Ly6^Chi^）	Obvious reduction	2014	[87]
	13F3	Murine melanoma model B16-BL6	MDSCs (CD11b^+^ CD11C^−^Gr1^+^)	Obvious reduction	2014	[87]
	HMBD-002	Murine colon cancer CT26 syngeneic CDX model	MDSCs (CD11b^+^Gr1^+^ MHCII^−^)	Obvious reduction	2022	[60]
	SG7	Murine breast cancer model 4T1	PMN-MDSCs (CD45^+^)	Obvious reduction	2020	[46]
	4M2-C12 IgG2a	Murine lymphoma model EL4	PMN-MDSC (CD45^+^CD11 b^+^Ly6G ^+^ Ly6C^lo/^)	Obvious reduction	2019	[88]
	4M2-C12 IgG2a	Murine colon cancer model CT26	PMN-MDSC (CD45^+^CD11 b^+^Ly6G ^+^ Ly6C^lo/^)	Obvious reduction	2019	[89]
	13F3	Murine oral squamous cell carcinomaora model 4MOSC1 and 4MOSC2	M-MDSCs (CD11b^+^LY6G^−^LY6^Chi^)	Obvious reduction	2023	[90]
Blocking VISTA together with TLR stimulation	VISTA-blocking mAb with TLR agonists CpG and R848	Murine melanoma mode lB16-BL6	M-MDSCs (CD11b^+^CD11C^−^LY6^Chi^LY6G^−^)	Both M-MDSCs and PMN-MDSCs (CD11b^+^CD11C^−^LY6C^int^ LY6G^+^) were reduced	2019	[51]
VISTA-targeted NIR-PIT with anti-VISTA-IR700	Anti-VISTA-IR700 followed by NIR light irradiation	Murine colon cancer model MC38	M-MDSC (CD45^+^CD11b^+^ Ly6C^+^Ly6G^−^)	Both M-MDSCs and PMN-MDSCs (CD45^+^ CD11b^+^ Ly6C^−^ Ly6G^+^) were reduced	2022	[88]
		Murine lewis lung carcinoma model LL2	M-MDSC (CD45^+^CD11b^+^ Ly6C^+^Ly6G^−^)	Both M-MDSCs and PMN-MDSCs (CD45^+^ CD11b^+^ Ly6C^−^ Ly6G^+^) were reduced	2022	[88]
A peptide vaccine and peptides derived from known tumor-associated antigens	TRP2 peptide	Murine melanoma model B16F10	M-MDSCs （CD11b^+^ Ly6C^hi^ Ly6G^−^）	Obvious reduction	2024	[78]
	Adpgk and Rpl18 peptide	Murine colon cancer model MC38	M-MDSCs (CD11b ^+^ Ly6C^hi^CD11c^neg^Ly6G^neg^)	Both M-MDSCs and PMN-MDSCs (CD11b ^+^ Ly6C^lo^ CD11c^neg^Ly6G^+^)were reduced	2024	[78]
VS3 and DVS3-Pal	peptide VS3 and peptide DVS3-Pal	Murine colorectal tumor model CT26	PMN-MDSCs (Cd11b^+^Ly6G^+^Ly6C^−int^)	Obvious reduction	2023	[91]

The bulk of the VISTA-targeted medications presently being tested in clinical and preclinical settings have demonstrated encouraging therapeutic effectiveness [92]. Current human mAbs targeting VISTA signaling pathway include but are not limited to, CI-8993 [93], KVA-12.1 [94], W0 180 [95], PMC-309 [96], SNS-101 [97], VS7 [98], VS147 [98], 4M2-C12 [89], and SG 7 [46].

Notably, the predominant MDSC subsets differ in murine tumor models which may lead to MDSC reduction discrepancies while targeting VISTA. In murine models, MDSCs were initially characterized as cells expressing both CD11b and Gr-1 markers. Gr-1 is a heterodimeric protein complex comprising subunits from the Ly6 family, and cells were further classified into M-MDSCs (Ly6C^+^) or PMN-MDSCs (Ly6G^+^) [99]. Subsequently, additional subpopulations of murine MDSCs have emerged, leading to the identification of additional cell-surface markers that define MDSCs. Currently, it is widely accepted that murine PMN-MDSCs are cells with neutrophil-like characteristics that express CD11b^+^Ly6^+^Ly6C^low^, while M-MDSCs are mononuclear cells defined as CD11b ^+^ Ly6Gl^ow/−^Ly6C^+^ [100]. However, relying solely on phenotypic criteria is inadequate for MDSC identification. Advances in techniques have led to the investigation of numerous MDSC subpopulations beyond the two broad categories [[101], [102], [103]]. Identification of MDSC subsets is critical in clinical settings, particularly in cancer immunotherapy, as these subsets exhibit varying biological characteristics in terms of T cell activation and immunosuppression, and can offer insights into managing immunotherapy resistance [104,105].

Of note, the suppressive pathways of PMN-MDSCs and M-MDSCs are distinct. The PMN-MDSC subset produces elevated levels of ROS that are unstable and active momentarily; as a result, they primarily exert immunosuppressive function by closely contacting T cells, disrupting T cells' binding to antigen-specific peptide-major histocompatibility complex (pMHC), but leaving them with the ability to respond to nonspecific stimulation [13,106]. Alessio Ugolini et al. also noted that PMN-MDSCs prevented DCs from directly presenting antigens while blocking antigen cross-presentation by these cells. The transmission of lipids is linked to this impact, which doesn't necessitate direct cell-to-cell contact [107]. In contrast, M-MDSCs preferentially generate nitric oxide (NO) and immunosuppressive cytokines like IL-10 and TGF-β, but they produce relatively little ROS, which results in generalized T-cell inactivation [100,108]. They both induce ER stress, activate transcription 3 (STAT3) expression, and produce arginase 1 [108]. In hyperoxia, M-MDSCs can quickly differentiate into TAMs, suggesting that they may have stronger suppressive activity than PMN-MDSCs [109,110]. Furthermore, despite the fact that MDSCs build up in both tumor tissues and peripheral lymphoid organs in tumor-bearing hosts, the evidence strongly suggests that tumor-infiltrated MDSCs exhibit a stronger suppressive feature than peripheral MDSCs [[111], [112], [113]]. During the development of the tumor, PMN-MDSCs gradually took the place of PMNs in the mice's spleens. Only tumors, where they manifest in the very early phases of the illness, contain activated PMN-MDSCs [114].

Whether VISTA inhibitors will maximally stop both PMN-MDSCs and M-MDSCs, however, is not certain. In contrast to the B16OVA model, Le Mercier et al. found that VISTA mAb treatment did not change the MDSC population infiltrating the MB49 bladder tumors. According to additional research, the majority of the MDSC community in the MB49 model is granulocytic phenotype, whereas the majority of the MDSC population in the B16OVA model is monocytic phenotype [87]. However, in 4T1 tumors, which have been shown to have substantial and uniform infiltrates of PMN-MDSCs, the active Fc form of the anti-VISTA antibody SG7 reduced the proportion of PMN-MDSCs. The PMN-MDSCs growth in the CT26, B16-BL6, and EL4 mice is suppressed by another anti-VISTA antibody, 4M2-C12 [89]. Additionally, Xu et al. discovered that TLR stimulation or VISTA-blocking mAb did not work to restore CD8^+^ T cells' capacity to produce IFN after being blocked by PMN-MDSCs [51]. This suggests that VISTA may control subpopulation-specific variations in MDSC migration and immunosuppression capacity. While M-MDSC function was reliant on IFN-γ signaling and independent of ER stress, Tcyganov et al. found that PMN-MDSC suppressive activity in cancer was controlled by the IRE1 and ATF6 pathways of the ER stress response [115]. Without affecting the suppressive action of PMN-MDSCs in spleens or tumors, deletion of IFN-R2 in M-MDSCs significantly decreased the expression of Nos2 in spleens [115].

The attachment of VISTA to PSGL-1 at acidic TME is disrupted by VS7, VS147, and SNS-101, which are pH-dependent [89,97,98]. It is notable that W0180 was shown to block VISTA's ability to bind to PSGL-1 regardless of the pH circumstances examined [116]. The anti-VISTA antibody HMBD-002 is an unusual IgG4 isotype that primarily targets the C-C′ loop, which is extremely conserved across model species. Across the spectrum of physiologically important pHs, HMBD-002 effectively reversed MDSC-mediated T-cell suppression and blocks VISTA-VSIG3 interactions [60]. A humanized monoclonal IgG1 antibody called 4M2-C12 has been demonstrated to block PSGL-1 and VSIG3 binding to VISTA in a dose-dependent way [89]. Purported associations between PSGL-1 and VSIG3 proteins are allegedly blocked by SG7.

Poor pharmacokinetics profiles, ineffectual tumor penetration, and expensive manufacturing expenses are some drawbacks of VISTA mAbs. Then came the development of small-molecule VISTA inhibitors, which had shortened half-lives and encouraged tumor infiltration [92]. The dietary antagonist CA170, the first small drug-like molecule inhibitor, has been applied in a dose-dependent way. In numerous tumor models in vivo, CA170 demonstrates anti-tumor effects akin to those of anti-PD-1 or anti-VISTA antibodies. Currently, a Phase 1 open-label, multicenter dose-escalation study (NCT02812875) of CA-170 has been completed for treating advanced solid tumors and lymphomas [17]. In patients with mesothelioma, CA-170 has demonstrated excellent safety characteristics with low rates of drug-related, immune-related or serious adverse events after oral dosing in continuous 21-day cycles. Treatment-emergent adverse event include but are not limited to, nausea, decreased appetite, anemia. As for efficacy, 7 of 11 evaluable patients had a best response of stable disease [117]. Moreover, CI-8993 has completed a Phase I trial (NCT04475523) evaluating its safety and anti-cancer efficacy in individuals with relapsed/refractory solid tumors [118], in which one patient experienced dose-limiting side effects related to cytokine release syndrome [119]. Another ongoing study (NCT05864144), a phase 1/2 open-label, multi-center trial, aims to assess the safety, tolerability, pharmacokinetics, pharmacodynamics, and efficacy of SNS-101 either alone or combined with cemiplimab in patients with advanced solid tumors [120]. AUPM 493 [121] and NSC622608 [122] are two other small-molecule VISTA inhibitors that have been created. VISTA-targeted near-infrared photoimmunotherapy (NIR-PIT) also reduced VISTA-expressing MDSCs in theTME of MC38-luc tumors in addition to anti-VISTA antibodies and small-molecule VISTA inhibitors [88].

The immunosuppressive ability of MDSC may be impaired by VISTA depletion in three ways: 1) by hindering the infiltration of immature myeloid progenitor cells; 2) by stopping MDSC recruitment; and 3) by lowering the number of MDSCs infiltrating the tumor.

**Fig. 4:**
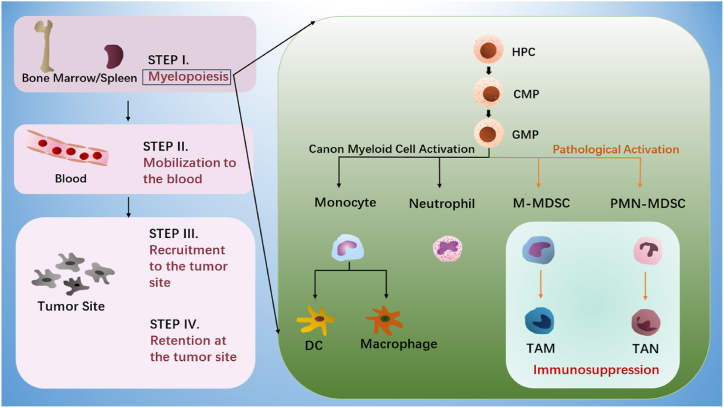
MDSCs distinction and enrollment. Four stages make up the novel MDSC development paradigm. The hematopoietic progenitor cell (HPC) develops into a common myeloid progenitor (CMP) and then a granulocyte-monocyte progenitor (GMP). During the first stage of amplified myelopoiesis, which takes place in the bone marrow and liver can differentiate into adult myeloid cells, such as neutrophils, monocytes, and dendritic cells (DC), and eventually into macrophages, during the process of classical myeloid cell activation. However, under conditions of long-term inflammation, GMP is pathologically activated and transforms into M-MDSC, which may transform into TAM, and PMN-MDSC, which may transform into TAN. In steps II and III, these myeloid cells move into the circulatory system, recruit to the tumor location, and stay there in steps IV.

Under physiological conditions, the differentiation of common myeloid progenitor (CMP) into neutrophils or monocytes, and subsequently into dendritic cells (DCs) and macrophages, is typical, while in cancer-induced myelopoiesis, it may lead to skewed differentiation into M-MDSCs and TAMs or PMN-MDSCs and tumor-associated neutrophils (TANs) (Fig. 4) [100]. A novel, multi-step approach has recently been put forth to clarify the creation and recruitment of MDSCs. This paradigm can be broken down into four steps: step I, which is myelopoiesis; step II, which is blood movement; step III, which is homing to the tumor site; and step IV, which is retention at the tumor site (Fig. 4) [123].

According to a research by Le Mercier et al. VISTA blockade decreased the tumor-infiltrating M-MDSCs in the B16 melanoma model without clearly deleting peripheral myeloid or T cell lineages [87]. This change indicates that VISTA mAb therapy may hinder the infiltration of immature myeloid progenitor cells, which may differentiate into MDSCs at the tumor site, to impair the recruitment of M-MDSCs into tumor issues, impairing the suppressive cellular signature of the TME [87]. The ability of VISTA inhibition to reduce the number of MDSCs in the tumor bed locally and increase the influx of tumor-specific effector T cells has been confirmed. This results in the development of an immune-stimulating TME and improved anti-tumor immunity [87,88].

## 3 Discussion

VISTA is an immune checkpoint protein with distinctive biological features and is shown to be another promising immune checkpoint treatment target. Beyond its role as a ligand or receptor that regulates naïve T cells, VISTA can also function as a soluble molecule released by human peripheral monocytes and Acute myeloid leukemia (AML) cells. This soluble form of VISTA may contribute to the impairment of immune cell cytotoxicity by enhancing the effects of galectin-9 and inhibiting granzyme B delivery [43,124]. Interestingly, VISTA seems to play a dual role in tumor development. While high VISTA expression is generally associated with poor prognosis in patients [125], it also has been found to correlate with improved survival in early-stage endometrial cancer and esophageal adenocarcinoma (EAC) patients [15,126]. The relevant mechanism may involve VISTA downregulating the anti-phagocytic signal SIRPα, which enhances the phagocytic capacity of M2 macrophages against cancer cells [127]. These conflicting findings highlight the necessity for further investigation into VISTA's complex role in TME. Moreover, although PSGL-1 and VSIG-3 have been confirmed as binding partners of VISTA, additional research is required to identify other potential counter-receptors.

MDSCs may play a role in predicting the immunosuppressive tumor milieu and regulating the outcomes of cancer treatment. In terms of cancer immunology, this heterogeneous population of myeloid cells exhibits fascinating biological characteristics and forms a complex network with the immune cells in the TME, for example, attracting Tregs, quickly differentiating into TAMs, and encouraging the differentiation of fibroblasts into cancer-associated fibroblasts (CAFs) [128].

The fact that VISTA is highly expressed on MDSCs casts light on its possible function as a regulator of MDSCs. Hypoxia and DNA methylation may help upregulate VISTA on MDSCs in the TME, which enhances VISTA association with its binding patterners in an acidic environment and results in the delivery of more co-inhibitory signals that suppress T cell response. Myeloid differentiation factor 88 (MyD88)-dependent Toll-like receptor (TLR) signaling on MDSCs is downregulated by VISTA, and VISTA inhibition greatly increases the production of TLR-mediated proinflammatory cytokines like IL-12, IL-6, GM-CSF, IFN-γ, MIP-1, and MIP-1. Additionally, VISTA-KO mice's MDSCs regularly displayed lower C5a receptor surface expression, which may increase the ability of MDSCs to inhibit antitumor immunity. To better understand the potential processes underlying this, more concrete proof is required.

Importantly, prior research has shown that VISTA-targeted therapies aid in decreasing MDSC infiltration in TME, which has paved the way for beating tolerance to current immune checkpoint drugs by targeting VISTA. Notably, there is ongoing debate over whether anti-VISTA drugs are able to suppress both PMN-MDSCs and M-MDSCs. There is an immediate need for more study to determine which MDSC subsets, if either, could be reduced by anti-VISTA treatment. The two major subpopulations of MDSC, PMN-MDSC and M-MDSC, may be regulated by VISTA in distinct ways.

The possible connections between VISTA and MDSCs are still being explored, and more research is needed to fully understand the precise methods VISTA uses to control MDSCs. Without a question, thorough research into the powerful immunotherapy target VISTA and one of the most popular immunosuppressive promoters, MDSC, will offer a way to combat immunotherapy resistance.

## Funding & acknowledgment

This study was funded by the Innovation and Development Joint Fund of Chongqing Natural Science Foundation (grant number CSTB2023NSCQ-LZX0099), Chongqing Science and Health Joint Medical High-end Talent Project (2022GDRC012), Science and Technology Research Program of 10.13039/501100007957Chongqing Municipal Education Commission (KJZD-K202100402), 10.13039/501100004374CQMU Program for Youth Innovation in Future Medicine (W0073).

## Ethics approval and consent to participate

Review and/or approval by an ethics committee are not needed for this study because this review does not involve clinical and basic experiments.

## Data availability

This study is a review and the raw data are available in referenced studies.

## CRediT authorship contribution statement

**Yayuan Deng:** Writing – review & editing, Writing – original draft, Data curation. **Mengjia Shi:** Writing – review & editing, Writing – original draft. **Lin Yi:** Writing – original draft, Methodology, Formal analysis. **Muhammad Naveed Khan:** Writing – review & editing, Investigation. **Zhijia Xia:** Writing – review & editing, Validation, Data curation. **Xiaosong Li:** Supervision, Resources, Funding acquisition, Conceptualization.

## Declaration of competing interest

All the authors declare that they have no competing interests.
